# Prevalence and Risk Factors for Atherosclerotic Cardiovascular Disease in 7704 Individuals: An Analysis from the Greek Registry for the Prevalence of Familial Hypercholesterolemia (GRegistry-FH)

**DOI:** 10.3390/jcdd11120411

**Published:** 2024-12-23

**Authors:** Genovefa Kolovou, Stamatis Makrygiannis, Christina Marvaki, Niki Pavlatou, Katerina Anagnostopoulou, Vasiliki Giannakopoulou, Georgios Goumas, Petros Kalogeropoulos, Vana Kolovou, Sotiria Limberi, Despina Perrea, Anastasios Tzenalis, Zeimpek Emre, Edison Jahaj, Zoi Kasiara, Ilias Giannakoulis, Ioannis Tsolakoglou, Olga Kadda, Nikolaos Tsaloukidis, Rafailia Koulaxidou, Aikaterini Marvaki, Stefanos Foussas, Andreas Melidonis, Giannis Hoursalas, Charalambos Vlachopoulos, Niki Katsiki, Haralampos Milionis, Evaggelos Liberopoulos, Helen Bilianou

**Affiliations:** 1 Lipoprotein Apheresis and Lipid Disorders Clinic, Cardiometabolic Center, Metropolitan Hospital, 18547 Athens, Greece; petros.kalo@gmail.com (P.K.); bkolovou@gmail.com (V.K.); 2 First Department of Cardiology, “Hygeia” Diagnostic and Therapeutic Centre of Athens, 15123 Athens, Greece; ssmakrygiannis@gmail.com; 3 Department of Nursing, University of West Attika, 12243 Athens, Greece; christina.marvaki@gmail.com; 4 Pathological and Surgical Nursing, University of West Attica, 12243 Athens, Greece; npavlatou@uniwa.gr; 5 Molecular Genetics and Genomics Department, Medical Neoscreen Ltd., 10431 Athens, Greece; kat_anag@yahoo.com; 6 Department of Cardiology, Tzanio Hospital, 18536 Piraeus, Greece; giannakopoulouvas@yahoo.gr; 7 Cardiology Clinic, Athens Euroclinic, 11521 Athens, Greece; ggoumasgr@yahoo.gr; 8 Department of Cardiology, Sotiria Hospital, 11527 Athens, Greece; sasalimberi@gmail.com; 9 Laboratory of Experimental Surgery and Surgical Research ‘N.S. Christeas’, National and Kapodistrian University of Athens Medical School, 11527 Athens, Greece; dperrea@lessr.eu; 10 Pathological Nursing/Intensive Care Unit, University of Patras, 26504 Patra, Greece; antzenalis@upatras.gr; 11 Radiology Department, Hospital of Xanthi, 67100 Xanthi, Greece; zeibemre@yahoo.gr; 12 Dermatology Department, Evangelismos General Hospital, 11635 Athens, Greece; edison.jahaj@gmail.com; 13 Department of Medicine & Department of Physical Education and Sport Science, Aristotle University of Thessaloniki, 57001 Thessaloniki, Greece; zoika1@outlook.com; 14 Argolidas General Hospital, 21200 Argos, Greece; egiann1966@gmail.com; 15 Department of Education, General Hospital of Thessaloniki ‘Agios Pavlos’, 55134 Thessaloniki, Greece; jtsolak@yahoo.gr; 16 Department of Electrophysiology and Pacing, Onassis Cardiac Surgery Centre, 17674 Kallithea, Greece; ol.kadda.31@gmail.com; 17 Department of Internal Medicine, University General Hospital ‘ATTIKO’, 12462 Athens, Greece; tsaloukidisn@gmail.com; 18 Biomedical Methods and Technology in Diagnosis, 12243 Athens, Greece; rafailiak17@gmail.com; 19 Department of Education, Katerini Hospital, 60100 Katerini, Greece; marvakikaterina@gmail.com; 20 Cardiology Department, Metropolitan General Hospital, 15562 Athens, Greece; sfoussas@gmail.com; 21 Diabetes Center, Metropolitan Hospital Athens, 15562 Athens, Greece; melidonisa@yahoo.com; 22 School of Medicine, European University Cyprus, 2404 Nicosia, Cyprus; ihoursal@otenet.gr (G.H.); nikikatsiki@hotmail.com (N.K.); 23 First Department of Cardiology, Hippokration Hospital, Medical School, National and Kapodistrian, University of Athens, 11527 Athens, Greece; cvlachop@otenet.gr; 24 Department of Nutritional Sciences and Dietetics, International Hellenic University, 57400 Thessaloniki, Greece; 25 Department of Internal Medicine, School of Medicine, University of Ioannina, 45110 Ioannina, Greece; hmilioni@uoi.gr; 26 First Department of Propaedeutic Internal Medicine, Medical School, Laiko General Hospital, National and Kapodistrian University of Athens, 11527 Athens, Greece; vaglimp@yahoo.com; 27 Independent Researcher, 4 Evkariou Street, 17122 Athens, Greece; elenib1@otenet.gr

**Keywords:** ASCVD (atherosclerotic cardiovascular disease), dyslipidemia, hypertension, prevalence, prevention, registry, risk factors

## Abstract

The intention of this study was to profile the cohort from the Greek Registry for the prevalence of Familial Hypercholesterolemia (GRegistry-FH) by estimating the prevalence of coronary artery disease (CAD), myocardial infarction (MI), stroke, dyslipidemia, arterial hypertension, diabetes mellitus (DM), pre-DM, smoking, abnormal thyroid function (ATF), and lipid values. The GRegistry-FH is a prospective study involving door-to-door interviews conducted by trained interviewers. Overall, 7704 individuals aged ≥18 years, randomly selected from all the regions of Greece, participated. The prevalence of atherosclerotic cardiovascular disease (ASCVD) was 13.9% (CAD 6%, MI 3.2%, stroke 4.7%). Treated hypercholesterolemia was present in 20.1%, arterial hypertension in 24%, and DM in 11.3% individuals (25.5% had pre-DM). The prevalence of smoking was 37.9% (29% current) and the prevalence of ATF was 13.1% (hypothyroidism 11.3%). A family history of ASCVD was reported by 60.5% (CAD 32.2%, stroke 28.3%). The mean (SD) lipid values in mg/dL were as follows: total cholesterol of 201.8 (41.5), low-density lipoprotein cholesterol of 126.3 (30.1), high-density lipoprotein cholesterol of 51.9 (12.5), and triglycerides of 135.9 (64.7). The GRegistry-FH highlights the significant prevalence of ASCVD and its risk factors among Greek adults, indicating a pressing need for early detection and management strategies to mitigate ASCVD burden. This nationwide registry serves as a crucial tool for guiding public health policies and personalized preventive measures (NCT03140605).

## 1. Introduction

Atherosclerotic cardiovascular disease (ASCVD) stands as the foremost cause of mortality in both women and men, with its prevalence surging amidst the escalating pandemics of obesity and cardiometabolic disorders [1,2,3]. Yet, 80% of ASCVD occurrences could be averted through prompt identification and proactive measures targeting modifiable risk factors such as elevated low-density lipoprotein cholesterol (LDL-C), hypertriglyceridemia, arterial hypertension, diabetes mellitus (DM), smoking, and obesity, among others [1]. Currently, ASCVD diagnosis relies heavily on symptom recognition, often occurring at an advanced disease stage and primarily managed by general practitioners, potentially leaving asymptomatic individuals untreated. This delay in intervention may exacerbate the risk of subsequent ASCVD events. Notably, conditions like familial hypercholesterolemia (FH) and untreated DM can significantly escalate ASCVD risk [4]. Early detection and intervention are thus imperative.

In Greece, where ASCVD prevalence, including FH, remains understudied, the Greek Registry for the prevalence of Familial Hypercholesterolemia (GRegistry-FH), initiated by the Hellenic College of Treatment of Atherosclerosis (HCTA), aims to address this gap. This study presents all the cohort profiles of 7704 participants from diverse Greek regions.

## 2. Methods

The rationale and design of the GRegistry-FH have been detailed elsewhere [5]. In the present analysis, the entire country was segmented into 14 regions (the Attica region was further divided into 8 regional units) according to the size of the population (based on the latest Hellenic General Population Census, Hellenic Statistical Authority, ELSTAT year 2011) [5]. Subsequently, an appropriate number of face-to-face trained interviewers were allocated to each region. The inclusion criteria encompassed individuals aged ≥18 and <80 years who were capable of providing informed consent while the exclusion criteria involved refusal to consent. Three hundred forty-nine individuals (339 men) refused to participate. Following data collection, a predetermined quota of fully completed questionnaires was meticulously inspected by the principal investigator. Two distinct files were generated, the first containing contact details of the participants, and the second encompassing all the provided information. Each case was assigned a unique identification number, facilitating the integration of anonymized data into a Statistical Package for Social Sciences (SPSS) file for subsequent analysis. The investigated measurements encompassed a comprehensive questionnaire probing demographic and behavioral characteristics, detailed medical history of ASCVD risk factors and events, as well as dietary and lifestyle habits of the participants. Demographic and clinical data were meticulously collected. These data included age, sex, height, body weight, body mass index (BMI: calculated by weight in Kg divided by squared height in m^2^), smoking status, family history of premature ASCVD, blood pressure (BP), and the values of key lipid and glucose parameters from recent biochemical tests, provided by the interviewees. All the equipment used by the interviewers to measure height, weight, and blood pressure were similar to ensure uniformity. The height was measured using a stadiometer (rounding to nearest cm), weight using calibrated electronic weighing scales (rounding to nearest kg), and blood pressure using the Omron m3 (Kyoto, Japan).

Additionally, the prevalence rates of various cardiovascular conditions such as coronary artery disease (CAD), myocardial infarction (MI), and stroke were assessed, alongside evaluations for hypercholesterolemia, hypertriglyceridemia, arterial hypertension, pre-DM, DM, and abnormal thyroid function (ATF). Thyroid function assessments were conducted to rule out secondary dyslipidemias. The definition of ASCVD encompassed a participant-reported history of stroke, CAD, MI, percutaneous coronary intervention, or coronary artery bypass graft. Positive family history criteria were established in accordance with 2019 ESC/EAS Guidelines [6]. Definitions for hypercholesterolemia (Total cholesterol > 200 mg/dL or LDL-C > 116 mg/dL), hypertriglyceridemia (>150 mg/dL), arterial hypertension (>140/90 mmHg), DM (blood glucose > 126 mg/dL), and pre-DM (blood glucose 100–125 mg/dL) were standardized based on established thresholds and/or medical treatment [6]. Smoking status was self-reported and categorized into current, ex-smoking, and never smoking. Abnormal thyroid-stimulating hormone (TSH) levels were delineated to identify hypothyroidism (≥4–4.5 mIU/L) and hyperthyroidism (≥0.4 mIU/L) based on specified TSH thresholds [7].

The GRegistry-FH was approved by Kapodistrian University of Athens Medical School (nr 1516016332/09.02.2016) and was given Trial registration number (NCT03395509).

Each participant gave written informed consent to participate in the study.

### Statistical Analysis

The current report involves descriptive statistics. The values of the continuous variables were expressed as the mean with standard deviation (SD) in brackets. Analysis was performed using IBM SPSS software version 26.0 (IBM Corp., Armonk, NY, USA).

## 3. Results

The characteristics of the study participants are provided in Table 1. The prevalence rates for ASCVD, treated hypercholesterolemia, arterial hypertension, DM, smoking, and ATF were determined as 13.9%, 20.1%, 24%, 10.6%, 37.9% (with 29% classified as current smokers), and 13.1%, respectively (Table 2). Of note, among the 5588 participants with relevant data, 25.5% had pre-DM. A notable 60.5% reported a positive family history of ASCVD. The lipid profile, blood pressure, and glucose values, all with and without therapeutic intervention, are summarized in Table 1. Specifically, untreated total cholesterol levels ≥200 mg/dL were prevalent in 33.7% (2411 participants), while untreated triglycerides levels ≥150 mg/dL were observed in a corresponding subset of 1135 subjects (14.8%).

## 4. Discussion

This study offers a comprehensive clinical and biochemical profile of participants enrolled in the GRegistry-FH across the entirety of Greece. Our findings underscore a noteworthy prevalence of individuals already with ASCVD or exhibiting one or more risk factors for ASCVD. Notably, historical epidemiological investigations such as the Seven Countries Study, conducted in the 1960s, delineated Greece, particularly the regions of Crete and Corfu, as possessing comparatively lower rates of CAD morbidity and mortality when contrasted with northern European nations and the United States [8]. In subsequent years, Greece underwent a notable shift away from the traditional Mediterranean diet coupled with high levels of physical activity, towards a Westernized dietary pattern, accompanied by a more sedentary lifestyle and elevated rates of smoking. Consequently, Greece has transitioned from its previous status as a region with low ASCVD risk to one where the burden of ASCVD has escalated significantly [6,9,10,11].

### 4.1. Prevalence of ASCVD

#### CAD/MI

In the nationwide GRegistry-FH, the prevalence of self-reported CAD/MI was 9.2% (CAD 6% and MI 3.2%). Comparative analyses with prior studies underscore the evolving landscape of ASCVD in Greece. For instance, the Saronikos study (2014) by Gikas et al. [12] documented CAD prevalence at 6.3% and MI at 3.6% among 2636 participants. Similarly, the EMENO study (2013–2016) by Stergiou et al. [13], with 4421 participants, found ASCVD prevalence at 5.8%. Moreover, the HYDRIA survey (2013–2014) revealed acute MI prevalence at 2.8% among 4011 participants [14]. Historical data from the Athens study [15] over 35 years ago indicated significantly lower CAD (1.6%) and MI (1.1%) rates, with a notable 1.9% of dubious CAD diagnoses. Over three decades after, our study highlights a striking 3.1-fold increase in CAD and MI rates, possibly attributed to heightened ASCVD risk factors such as obesity, pre-DM, and DM, alongside advancements in diagnostic sensitivity. Conversely, Western nations have witnessed declining CAD prevalence and mortality, attributed to smoking reduction, as well as more effective LDL-C and blood pressure management [16,17,18,19,20]. The Seven Countries Study further elucidates these trends, demonstrating declining CAD mortality in some cohorts while noting increases in others [20], including Greece, underlining the critical role of comprehensive nationwide registries for ASCVD management in dynamically evolving populations like Greece, recently reclassified from low- to medium-risk country status [11].

### 4.2. Stroke

In the nationwide GRegistry-FH, the prevalence of self-reported stroke was documented at 4.7%. A comparison with the HYDRIA survey (2013–2014) by Kanellou et al. [13], comprising 4011 participants, revealed a lower stroke prevalence of 1.9%. Notably, advanced age significantly influenced stroke occurrence, with individuals aged 65 years and above exhibiting over a 4-fold higher prevalence compared to those below 65 years (1% vs. 4.7%, respectively). This age-related discrepancy underscores the heightened vulnerability of older adults to stroke, reflecting the well-established association between aging and increased cerebrovascular risk [20,21]. Such epidemiological insights are crucial for targeted intervention strategies and resource allocation, particularly in aging populations where stroke burden disproportionately impacts healthcare systems and public health initiatives.

### 4.3. Family History of Premature ASCVD

In the nationwide GRegistry-FH, significant rates of family history of premature ASCVD emerged, i.e., 32.2% for CAD and 28.3% for stroke. Comparatively, the PHAETHON study [22], encompassing 800 acute coronary syndrome Greek patients discharged from hospitals during 2012–2014, noted a family history of premature CAD in 25.8% of the cases. Moreover, the global CLARIFY study, with over 33,000 CAD patients across 45 countries, reported a premature family history rate of 28%, escalating to 35% among 559 Greek patients. Ambroziak et al. [23] found that younger MI patients (<50 years) had a higher incidence of premature ASCVD among first-degree relatives compared to older MI patients and non-MI individuals of the same age group. Furthermore, Rasooly et al.’s population-based investigation [24] highlighted that over 44% of the US participants had a family history of premature ASCVD and/or DM, emphasizing its significance in risk assessment. These findings underscore the pivotal role of high-risk family history in identifying individuals susceptible to ASCVD, necessitating robust preventive strategies for early detection and intervention. Recognizing the impact of familial predisposition to ASCVD enables the reclassification of at-risk individuals into higher-risk categories, aligning with guidelines for enhanced clinical management and public health initiatives [5].

### 4.4. Prevalence of Treated Hypercholesterolemia

In the nationwide GRegistry-FH, the documented prevalence of treated hypercholesterolemia stood at 20.1%. Comparable studies, such as the EMENO investigation by Stergiou et al. [13], reported a strikingly high burden of dyslipidemia among Greek adults, with over 50% exhibiting some form of dyslipidemia, predominantly characterized by elevated total cholesterol levels exceeding 200 mg/dL, with only 14.1% of them utilizing lipid-lowering medications. Moreover, findings from the Saronikos study [12] revealed a dyslipidemia prevalence of 24%, while the Hellenic National Nutrition and Health Examination Survey (HNNHS) [25] conducted during 2013–2015 indicating that approximately 20.7% of Greek adults were affected by dyslipidemia, with a noteworthy 59.0% taking hypolipidemic drug treatment. These observations collectively underscore the substantial burden of dyslipidemia in the Greek population, highlighting the imperative for comprehensive strategies aimed at the prevention, early detection, and effective management of this significant cardiovascular risk factor. There has been an effort to address this need in the recently published Hellenic Atherosclerosis Society (HAS) guidelines for the diagnosis and treatment of dyslipidemias [26].

### 4.5. Prevalence of Arterial Hypertension

The documented prevalence of arterial hypertension stood at 24%. Contrasting findings from a survey conducted in a small rural Greek village in 1999 by Stergiou et al. [13] showed a lower prevalence of arterial hypertension, approximately 7% less than the one observed in the GRegistry-FH. Conversely, the Saronikos study [12] reported a higher prevalence of arterial hypertension at 27.2%, while the EMENO study [13] documented an even higher prevalence of 39%. Similarly, the HYDRIA survey [14] reported a prevalence of arterial hypertension at 41.7%. Notably, the GRegistry-FH, encompassing a significantly larger and more representative sample across all the regions of Greece, demonstrated a prevalence consistent with broader Westernized countries, where arterial hypertension rates typically range from 24% to 38% [27]. These disparities underscore the complex interplay of various factors contributing to arterial hypertension prevalence, emphasizing the need for targeted public health interventions tailored to specific population contexts.

### 4.6. Prevalence of Pre-DM

We observed a prevalence of 25.5% for pre-DM. In a study by Makrilakis et al. [28] based on data from the EMENO study (4393 individuals) the reported prevalence of known DM was 10.4%, with pre-DM observed in 12.4% of the individuals. These findings present a noteworthy contrast to our registry’s figures, indicating a notably higher burden of pre-DM in our study cohort. Such disparities underscore the multifactorial nature of pre-DM prevalence, warranting further investigation into the underlying determinants and potential implications for preventive health strategies.

### 4.7. Prevalence of DM

The prevalence of DM in the nationwide GRegistry-FH was 10.6%, with 25.5% of the 5588 participants having pre-DM. Comparable figures were observed in the Saronikos study (11.1%) [12] and the EMENO study (11.7%) [13], as well as in the HYDRIA survey [14], where self-reported DM prevalence was 11.4%. Over the last two decades, there has been a notable escalation in DM prevalence in Greece, rising from 8.2% in 2002 to 11.1% in 2016 [29,30]. This trend aligns with findings from the ATTICA study [31], which attributed this increase to factors such as obesity, population aging, and genetic predisposition (e.g., family history of DM). When compared to other Mediterranean nations, Greece exhibits one of the highest reported rates of known DM, with figures ranging from 5.5% in France (2012) [32] to 9% in Turkey (2010) [33].

### 4.8. Prevalence of Smoking

The prevalence of smokers in the GRegistry-FH was 37.9% (current smokers accounting for 29%). Notably, smoking prevalence in Greece ranks among the highest globally, posing a significant public health concern [34]. Encouragingly, our registry indicates a declining trend in current smoking prevalence over recent decades, dropping from 40.1% in 2006 [30] to 38.9% in 2014, and further to 29% in our study. However, findings from Filippidis et al. [34] suggest a more stable trend, with no significant change in smoking prevalence between 2006 and 2010 (43.1% to 42.6%). Current smoking rates in the Saronikos [12] and EMENO studies [13] were notably higher compared to our findings, standing at 38.9% and 39%, respectively, underscoring the variations across different populations. Similarly to our registry, the HYDRIA survey [14] reported a lower smoking prevalence of 31.6%. Over the past 15 years, Greece has implemented legislative measures and initiatives aimed at curbing tobacco consumption, reflecting ongoing efforts to address this pressing public health issue [35].

### 4.9. Values of Total Cholesterol, LDL-C, HDL-C, and Triglycerides

**Figure 1:**
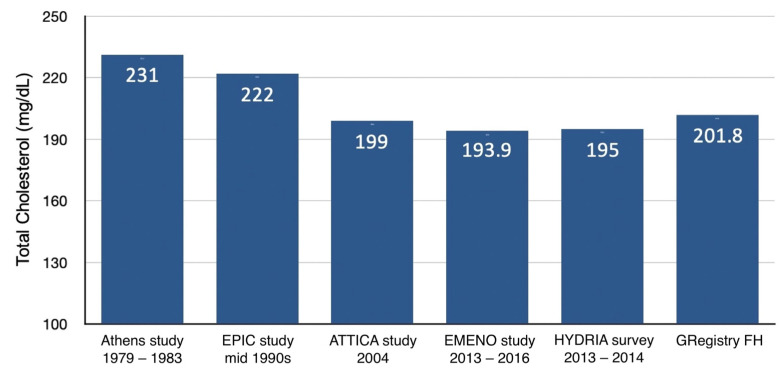
Total cholesterol values reported in different Greek population studies.

Traditional lipid values, including total cholesterol, LDL-C, HDL-C, and triglycerides, are crucial determinants in the pathogenesis and progression of ASCVD. Therefore, assessing these lipid parameters in the general population is of paramount importance for understanding cardiovascular risk at a national level. Benetou et al. [36] conducted the EPIC study, a prospective cohort involving 11,645 participants from across Greece during the mid-1990s. Their findings revealed a mean total cholesterol level of 222 mg/dL. However, direct comparison with the GRegistry-FH results is cautioned due to methodological disparities, as the EPIC study relied on volunteer participants and may not be fully representative of the Greek population (Figure 1).

Panagiotakos et al. [37] presented lipid profiles of individuals from the Attica region. The total cholesterol values among the GRegistry-FH participants were 201.8 (41.5) mg/dL, slightly elevated but comparable with those in the ATTICA study. A more prominent difference was noted in comparison with those observed in the EMENO study [13] and the HYDREA survey [14] which reported 193.9 (44.4) mg/dL and 195.0 (39.2) mg/dL, respectively (Figure 1). These findings suggest a concerning trend of increasing dyslipidemia prevalence in Greece over the past decade, likely influenced by various behavioral factors such as urbanization, dietary habits, physical inactivity, and the socioeconomic crisis experienced in recent years, which has adversely impacted healthcare services and public health initiatives.

### 4.10. Prevalence of Hypercholesterolemia

The prevalence of participants with total cholesterol levels ≥200 mg/dL varies across different studies. In the ATTICA study conducted by Panagiotakos et al. [37], this percentage was reported as 43%, while in the EMENO study by Stergiou et al. [13], it was slightly lower at 42.7%. Similarly, the HNNHS study by Magriplis et al. [25], found a prevalence of 38.6%, whereas in the GRegistry-FH, it was 33.7%.

Regarding lipid-lowering therapy, there is variability in the reported rates across studies. A decade ago, the EMENO study reported a prevalence of 14.1% [13], which has since increased to 20.1% in the GRegistry-FH and 20.5% in the HYDRIA survey by Kanellou et al. [14]. Therefore, the data from the GRegistry-FH provide reassurance that the prevalence of hypercholesterolemia has not increased significantly in Greece over the last decade, whereas there has been a notable rise in the utilization of lipid-lowering therapy.

### 4.11. Prevalence of Hypertriglyceridemia

In the GRegistry-FH, the prevalence of hypertriglyceridemia was 14.8%. The mean triglyceride level was 135.8 (64.7) mg/dL, a value notably higher than that reported in the HNNHS study (83 mg/dL) by Magriplis et al. [25], as well as in other countries such as the USA (National Health and Nutrition Examination Survey [NHANES], 103.5 mg/dL) [38] and France (Esteban Study, 104.5 mg/dL) [39]. In the EMENO study [13], hypertriglyceridemia was found in 27.8% of the participants, comparable to the NHANES data reporting 24.2% prevalence of hypertriglyceridemia. Previous Greek studies, such as the HNNHS [25] and the ATTICA [37], reported lower rates of hypertriglyceridemia at 13.9% and 20.4%, respectively. Notably, the DYSIS (Greek Dyslipidemia International Study) in 2012 [40] revealed a high prevalence of hypertriglyceridemia at 39.9% among dyslipidemic patients despite statin treatment. These findings underscore the significant burden of hypertriglyceridemia in Greece, suggesting potential implications for targeted interventions and management strategies.

### 4.12. Prevalence of Thyroid Dysfunction

In the GRegistry-FH, 13.1% of the participants presented with thyroid dysfunction, predominantly hypothyroidism. Comparatively, the prevalence of hypothyroidism in the general population varied between 0.3% and 3.7% in the USA and between 0.2% and 3.5% in Europe, contingent upon the specific diagnostic criteria used [41,42]. A comprehensive meta-analysis spanning nine European countries (Denmark, England, France, Germany, Italy, Norway, Scotland, Spain, and The Netherlands) was conducted [42]. According to this meta-analysis, nearly 11% of Europeans have thyroid dysfunction, and of special concern, only about half of them are aware of their condition. These findings highlight the notable burden of thyroid dysfunction observed in the GRegistry-FH cohort, underscoring the importance of comprehensive thyroid screening and management strategies within the general population. In the EMENO study [13], the prevalence of thyroid diseases was 9%; in the HUNT study [43], it was 9% in women and 3% in men.

### 4.13. Limitations

Since the present analysis does not attempt to establish causality, the main disadvantage of this study design is not relevant to this work. However, a cross-sectional design should preferably have a limited time frame for data collection and in our case the scheduled duration was extended due to the COVID-19 pandemic. Therefore, temporal changes may have affected the observed prevalences.

Effort has been made to recruit a representative sample, by following the geographical distribution of the official national census and avoiding enrolling in any kind of healthcare provision facilities. Nevertheless, the mandatory exclusion of persons that decline consent may have affected the representativeness of the sample. It seems that women tended to be more willing to participate, so the overall prevalence of any variables that are known to have gender differences must be cautiously appraised. A gender-based analysis of this cohort data will follow to further elucidate any differences.

The dependence on self-reported data for ASCVD risk factors, events and family history may introduce recall bias, potentially affecting the accuracy of reported rates. However, it should be noted that biochemical data, medical reports, and prescriptions were also reviewed and recorded, if available, thus minimizing the biases. Furthermore, there is some evidence showing that self-reported data concerning well-known ASCVD risk factors may be reliable, e.g., for DM, arterial hypertension, and dyslipidemia [44]. Similarly, the validity of self-reported cardiac events including diagnosed MI, angina, percutaneous coronary intervention, and coronary bypass surgery is likely to be very high [45]. Of course, as in similar studies, the prevalence rates reported in the current analysis may underestimate the true burden of ASCVD and related risk factors due to undiagnosed cases or individuals not seeking medical advice/care.

Despite the above limitations, the GRegistry-FH provides important data concerning the prevalence of ASCVD and ASCVD risk factors in the general adult population of Greece, to inform current clinical practice, policy making and future research targeting ASCVD. It also provides a solid base for comparisons with relevant observations in other populations.

## 5. Conclusions

The GRegistry-FH has provided a comprehensive cohort profile of 7704 participants across Greece, offering valuable insights into the prevalence and distribution of ASCVD and its risk factors. The present data reveal a significant presence of ASCVD and associated risk factors within the Greek adult population, indicating a critical need for early detection and preventive measures. Notably, this study shows a prevalence of ASCVD at 13.9%, with treated hypercholesterolemia at 20.1%, arterial hypertension at 24%, and DM at 10.6%. Additionally, 25.5% of the participants were found to have pre-DM, and 37.9% were smokers, highlighting the widespread nature of these risk factors.

Comparisons to historical data and other regional studies indicate a notable increase in ASCVD prevalence and its risk factors over recent decades, likely driven by lifestyle changes, such as a shift from the traditional Mediterranean diet to a more Westernized diet, and higher rates of sedentary behavior and smoking. For instance, the study observed a 3.1-fold increase in CAD and MI rates compared to data from over 35 years ago.

A family history of premature ASCVD also emerged as a significant risk factor, underscoring the importance of including familial patterns in risk assessments. The findings align with global trends that show a decline in CAD prevalence in some industrialized countries due to improved preventive measures and treatments, yet Greece has experienced an opposite trend, reflecting the urgent need for enhanced public health strategies.

The GRegistry-FH study emphasizes the critical need for continued monitoring, early diagnosis, and effective management of ASCVD risk factors in Greece. Public health initiatives should focus on promoting healthier lifestyles, improving awareness, and providing targeted interventions to reduce the burden of ASCVD. The registry serves as an essential tool for guiding these efforts, aiming to mitigate the rising trend of ASCVD and improve cardiovascular health outcomes in the Greek population.

## Figures and Tables

**Table 1:** Characteristics of study participants.

Characteristics of Study Participants (*n* = 7704)	
Characteristics	
Age (years)	49.7 (16.9)
Hight (cm)	169.2 (9.2)
Weight (kg)	76.0 (21.2)
Men/women (n)	3262/4442
Men age (years)	51.0 (17.1)
Women age (years)	48.7 (16.9)
Medical history	
Coronary artery disease	6.0%
Myocardial infarction	3.2%
Stroke	4.7%
Treated hypercholesterolemia	20.1%
Atrial hypertension	24.0%
Diabetes mellitus	10.6%
Current smoking	29.0%
Abnormal thyroid function	13.1%
Family history of coronary artery disease	32.2%
Family history of stroke	28.3%

Continuous variables are expressed as mean (SD); SD = standard deviation.

**Table 2:** Investigated measurements.

Investigated Measurements	
Variable	
Systolic blood pressure (mmHg)	123 (17)
Diastolic blood pressure (mmHg)	75 (11)
Blood glucose without treatment (mg/dL)	99.3 (30.0)
Total cholesterol without treatment (mg/dL)	201.8 (41.5)
HDL-C without treatment (mg/dL)	51.9 (12.5)
LDL-C without treatment (mg/dL)	126.3 (30.2)
Triglycerides without treatment (mg/dL)	135.9 (64.7)

HDL-C = high-density lipoprotein; LDL-C = low-density lipoprotein; values are expressed as mean (SD).

## Data Availability

Data are contained within the article.
